# Handball-specific loading acutely reduces the acromiohumeral distance in experienced handball players and in non-handball experienced athletes

**DOI:** 10.3389/fspor.2022.997401

**Published:** 2022-09-16

**Authors:** Carolin Rentz, Kirsten Legerlotz

**Affiliations:** Institute of Sport Science, Humboldt University of Berlin, Berlin, Germany

**Keywords:** shoulder, handball, ultrasonography, acromiohumeral distance, impingement syndrome

## Abstract

**Context:**

When playing handball, the preservation of the subacromial space, which can be quantified by the acromiohumeral distance (AHD), plays a crucial role for shoulder health of handball players. Acute effects of handball-specific loading on the subacromial space with consideration of individual adaptions resulting from long-term handball-specific loading experience have yet to be determined in order to prevent injuries such as e. g. an impingement of the supraspinatus tendon.

**Objective:**

To (1) assess the acute effects of handball-specific loading on the AHD in healthy experienced handball players (HB) and non-handball experienced athletes (CG) and (2) to assess the AHD behavior in relation to individual intrinsic factors to identify possible risk factors and the effect of handball-specific experience associated adaptations.

**Participants:**

20 HB (10m; 10f) and 20 CG (10m; 10f); 24 ± 5 years.

**Intervention:**

Handball-specific loading protocol.

**Main outcome measures:**

The AHD was measured by ultrasonography at 0° and 60° abduction pre and post intervention. Isometric shoulder strength was measured with hand-held dynamometry. Shoulder range of motion (ROM) was measured with goniometry.

**Results:**

Handball-specific loading led to significantly reduced AHD in the dominant shoulder in the 60° abducted position in both groups (HB: −1.7 ± 2.0 mm; *p* = 0.001, d = 0.69; CG: −1.1 ± 2.0 mm; *p* = 0.024, d = 0.37) and in the non-dominant shoulder in 0° (−0.7 ± 1.5 mm; *p* = 0.038, d = 0.35) and 60° abducted position (−1.3 ± 1.8 mm; *p* = 0.004, d = 0.69) in HB only. Handball-specific loading enhanced AHD reduction when elevating the shoulder from 0° to 60° in both groups and arms. Larger shoulder abduction strength affected the maintenance of the AHD positively. HB demonstrated less shoulder strength compared to CG, while ROM did not differ.

**Conclusions:**

Handball-specific loading can affect the ability to preserve the subacromial space which might put handball players at risk for shoulder injuries. Poor shoulder strength can aggravate this mechanism. Therefore, implementation of strengthening exercises of the external rotator and abductor muscles in the training schedule may improve shoulder health of handball players.

## Introduction

Handball is a physically demanding sport, characterized by a large number of sprints, jumps and throws, and quick changes in direction. While those characteristics contribute to its huge international popularity (1) the repetitive high-velocity overhead motions are associated with a high prevalence of injuries (2). Beside acute traumatic injuries (3) occurring in relation to physical playing techniques or contact with the opponent, chronic overuse injuries (4) frequently occur on account of repetitive loading imposing high stresses on tissues of the upper extremity (5, 6). In handball, the shoulder joint is the joint most affected by chronic shoulder pain and overuse injury (7). To address the high prevalence of shoulder injuries in handball, mechanisms and risk factors leading to those injuries need to be identified and investigated across various competition levels and in both sexes.

Apart from many other shoulder structures, pathologies of the rotator cuff and the subacromial bursa are considered to be a principal cause of shoulder pain (8). These structures are located inside of the subacromial space and are known to be structurally damaged when this space is reduced, posing a risk for a subacromial impingement syndrome (9). The quantification of the subacromial space by measuring the linear distance between the acromion and the humeral head, which is called the acromiohumeral distance (AHD), has been established with various radiological methods (10, 11). AHDs in shoulders of asymptomatic participants, measured with ultrasonography in a neutral shoulder position, generally vary between 10 and 15 mm, while values below 7 mm indicate pathology (9, 12, 13). The subacromial space has been shown to become smaller during shoulder abduction and elevation (11, 14, 15). As frequently performed handball-specific motions include shoulder abduction and external rotation during throwing, passing and specific defense techniques, these movements may lead to periodically reduced subacromial spaces in handball players (16). In conclusion, the preservation of the subacromial space especially in overhead athletes appears to be crucial to prevent an impingement of the rotator cuff tendons (17).

It has yet to be determined which factors contribute to maintaining this space and whether handball players can do this better by means of their specific adaptations. It is known that the population of overhead athletes whose shoulders are exposed to specific and chronic overhead loading displays biomechanical and structural adaptive changes such as altered shoulder range of motion (ROM) patterns and altered strength ratios in the dominant shoulder (16, 18). Furthermore, handball-specific loading may lead to altered shoulder strength or strength ratios, represented by an enhanced internal rotation strength and reduced external rotation strength (19).

Several previous studies have investigated the acute effects of exercise induced fatigue on several parameters related to the shoulder joint of athletes (20) and especially of handball players (21, 22). An unspecific shoulder-muscle fatiguing protocol in overhead athletes has been shown to increase the AHD, whereby the scapular behavior was suspected to provide a protective compensating and impingement sparing situation (23). However, the acute effects of handball-specific loading on the subacromial space have not yet been investigated. Furthermore, it is not known how individual adaptions as a consequence of long-term handball-specific loading experience affect the preservation of the subacromial space in response to acute handball-specific loading.

The aim of this study was to assess the acute effects of handball-specific loading on the AHD in experienced handball players and in non-handball experienced athletes. Handball-specific loading during gameplay was simulated by means of a standardized protocol of handball-related movements to investigate its effects on the subacromial space. We hypothesized that handball-specific loading will reduce the AHD.

As a secondary objective, we examined the AHD behavior in relation to individual intrinsic factors such as shoulder strength and range of motion in order to identify possible intrinsic risk factors which could be addressed by preventive interventions, and which may contribute to the development of suitable and sport-specific prevention programs. We hypothesized that poor shoulder strength and poor range of motion will have a negative effect on the ability to preserve the subacromial space.

Third, we investigated the effect of handball-specific experience respectively therewith associated adaptations on the response of the AHD to handball-specific loading by comparing experienced with non-experienced handball players. We hypothesized that handball-specific experience and therewith associated adaptations may affect the response of the AHD to handball-specific loading while we did not expect an effect of sex.

## Materials and methods

### Study design and participants

A group of experienced handball players (HB; *n* = 20; 10 m and 10 f) were recruited from local handball clubs by contacting the coaches via email while a control group of non-handball experienced athletes (CG; *n* = 20; 10 m and 10 f), were recruited from sport science students attending handball courses as part of their sport science degree by contacting the handball lecturer of an university. To be included in the study, all participants had to be between 18 and 40 years of age. Participants of the HB group had to have played handball at competition level (no goalkeeper) with at least two training sessions per week and a minimum of 5 years' experience. Participants of the CG were physically fit sport science students. They had learned and practiced handball specific movements in the handball course for several weeks but had no further previous handball experience other than in the one acquired in the University course. Exclusion criteria were current shoulder pain and shoulder pain during 6 months before the study for which a medical doctor was consulted. Furthermore, people with systemic diseases, previous shoulder surgery as well as diagnosed and documented structural damage or known anatomical alterations of the shoulder joint were excluded from the study.

The participants took part in two experimental sessions: (1) patient-reported outcome measures were obtained and AHD was measured with ultrasonography before and after a handball-specific loading protocol. (2) anthropometric measures, shoulder muscle strength and range of motion were obtained. All athletes were assessed within the same phase of the competitive season to prevent varying conditions within the course of the season.

The sessions took part at the sports facilities of the handball clubs (HB group) and at the University sports facility (CG), respectively. Measurements were performed by the same two trained investigators, of which one is a physiotherapist and one is a sports scientist. The study protocol was approved by the Ethics Committee of the Faculty of Humanities and Social Sciences of the Humboldt-Universität zu Berlin (HU-KSBFEK_2019_0013).

### Anthropometric data assessment and patient-reported outcome measurement

Anthropometric measures such as body height, body mass, arm (humeral head – ulnar styloid process) and forearm (lateral epicondyle – ulnar styloid process) length were determined. The dominant arm was defined as the arm with which the participants reported to preferably throw a ball. To identify shoulder impairments all participants completed the German version of the Kerlan-Jobe orthopedic clinic shoulder and elbow score (KJOC-G), which is a reliable and valid tool that specifically identifies impairments, activity limitations and sports participation restrictions of overhead athletes (24).

Participants underwent clinical examination, including active movements and impingement tests (Hawkins, Neer, and Jobe tests) (25), by an experienced physiotherapist. Reliability and diagnostic accuracy for these tests were confirmed by a previous study (26). Participants with two out of three positive tests were excluded from the study. Currently existing shoulder pain intensity was monitored using the 11-point numeric rating scale (0 = no pain, 10 = pain as bad as it could be) (27).

### Assessment of isometric shoulder strength

The participants were advised not to intensively exercise 48h before examination. Abduction (ABD), external rotation (ER) and internal rotation (IR) maximum isometric shoulder strength of the dominant and non-dominant shoulder were measured with a microFET2 hand-held dynamometer (Hoggan Health Industries Inc., West Jordan, UT, USA) as described previously (11). Hand-held dynamometry is regarded as valid and reliable tool for the assessment of shoulder strength (28, 29). Three repetitions were performed for each arm and muscle group and the mean was calculated. Peak force values in Newton (N) were converted to torque (Nm) by multiplying the force values by the length of the respective lever arm, which was the forearm in ER- and IR-strength measurement and the full arm in ABD-strength measurement. In addition, joint torques were normalized to body weight (Nm/kg) (30). The ratio of peak isometric external- to internal-rotation muscle force (ER/IR ratio) was calculated. The subsequent group and correlation analyses were performed with the normalized torques.

### Assessment of shoulder range of motion

The active internal (IR) and external rotation (ER) of the dominant and the non-dominant shoulder joint were measured as described previously (11) with goniometry (Digital goniometer Baseline^®^ Absolute Axis 360 Grad-Digital-Goniometer, model 1013990) which is considered as reliable and valid method for shoulder mobility measurements (31). The total range of motion (TROM) was calculated by the sum of IR and ER (32). Furthermore, the glenohumeral internal rotation deficit (GIRD), defined as difference between IR of the dominant shoulder and IR of the non-dominant shoulder, and external rotation gain, defined as difference in ER between both sides, were calculated (33, 34).

### Ultrasound measurement

Sonographic measurements were performed before (PRE) and after (POST) execution of the handball-specific training protocol, using the mobile Echo Blaster 128 CEXT (Telemed Ltd, Vilnius, Lithunia) and a 5.0- to 8.0-MHz linear transducer (LV7.5/60/128Z-2). The participants were sitting upright during the measurements. Images were taken for the dominant and the non-dominant shoulder in two standardized positions: (1) 0° shoulder neutral position with the arm positioned aside the body; (2) 60° of active abduction in the coronal plane. The 60° abduction angle was determined with a digital goniometer. A marker tape was placed on an adjacent wall at the level of the participant's finger tips in order to maintain the correct angle of arm abduction during the measurement. The participant's arm was moved back into the neutral position in between measurements to avoid fatigue. Each position was repeated two times for every shoulder, and the mean was calculated.

The AHD was measured in the coronal plane by placing the ultrasound transducer on the center of the acromion parallel to the longitudinal axis of the humerus as described previously (11). The minimal detectable change (MDC) is 0.02 mm in neutral position (0°) as determined previously (11). All measurements were performed by a single investigator, who is a licensed and musculoskeletal ultrasonography trained physiotherapist. The ultrasound images were analyzed by quantifying the shortest distance between the inferior edge of the acromion and the most superior aspect of the humerus (Image J 1.32 software).

The reliability of the applied measurement methodology was verified in a preliminary study, with an excellent intra-rater reliability (ICC_3, 1_ 0.996) and inter-rater reliability (ICC_2, 1_ 0.997) (11).

Ultrasound images were excluded from statistical analyses when accurate distance measurements with the software were impossible due to poor image quality or in case the respective landmarks of the image could not be clearly visualized. In total, for all measurements 15 of 320 values could not be established.

### Handball-specific loading

Literature on sport-specific characteristics of a handball game (5, 35, 36) as well as previous studies applying handball-specific intervention protocols (21, 37) were reviewed in order to mimic authentic game specific handball load. The newly developed handball-specific loading protocol consisted of a standardized warm-up with a general and a handball-specific part, followed by a series of handball-related movements including repetitive passing, throwing, tackling and blocking (Supplementary material). It lasted 30–45 mins, depending on individual performance, and was conducted with a team handball size 2 for female participants as well as male non-handball experienced participants and size 3 for experienced male handball players. It was performed in sports facilities with a handball court (size 20 × 40 m) and a standard handball goal (size 2 × 3 m). A goalkeeper, who was not part of the study group, was in the goal to ensure an authentic setting during the shots on goal. All exercises were performed pairwise, instructed by the respective team coach or a handball experienced member of the study team, who supervised the loading protocol, ensuring that it was conducted as planned. The rate of perceived exertion was evaluated as described by Borg (38). A Scale (6 to 20) indicated the subjectively perceived level of local fatigue or exertion in the throwing arm and the non-throwing arm during the handball-specific protocol.

### Statistical analysis

All statistical analyses were performed using IBM SPSS Statistics software for Mac, Version 24.0 (Armonk, NY: IBM Corp). Data normality was assessed by the Shapiro-Wilk test and frequency histograms. The significance level (α) was set at *p* ≤ 0.05 for all statistical procedures.

Group and sex differences in demographic characteristics, ROM, strength and AHD were analyzed by independent *t*-tests. Differences between the shoulder sides and between pre and post AHD measurement after handball-specific loading within the groups were analyzed by paired *t*-tests. Mann-Whitney-U-Test and Wilcoxon signed-rank test, respectively, were used in case of non-normally distributed variables (HB: Age, KJOC-G score dominant and non-dominant; CG: KJOC-G score dominant and non-dominant, ER/IR strength ratio dominant).

A repeated measures ANOVA was used to detect between-group differences and interactions between group (HB and CG) and time (pre and post).

To assess associations between ROM, normalized peak torque and ER/IR strength ratios and AHD and AHD changes, respectively, bivariate correlation analyses by calculating Pearson's correlation coefficients (r) and non-parametric Spearman's rank correlation coefficients (r_s_), for non-normally distributed variables were used. Classifications were used as follows: <0.10 = negligible, 0.10–0.39 = weak, 0.40–0.69 = moderate, 0.70–0.89 = strong and >0.90 = very strong correlation (39). Comparisons of correlations were performed according to Lenhard and Lenhard (40).

## Results

The demographic characteristics did not differ significantly between HB and CG (Table 1).

**Table 1:** Demographic and sports-related characteristics of the participants.

	**All participants**	**Handball players**	**Control group**
Number	40 (100%)	20 (50.0%)	20 (50.0%)
Sex (m/f)	20/20 (50.0/50.0%)	10/10 (50.0/50.0%)	10/10 (50.0/50.0%)
Age, y	23.65 ± 4.7	24.1 ± 4.3	23.2 ± 5.2
Height, cm	172.6 ± 8.2	172.4 ± 7.7	172.9 ± 8.9
Body weight, kg	72.4 ± 12.2	72.3 ± 13.6	72.4 ± 11.0
BMI, kg/m^2^	24.2 ± 2.8	24.2 ± 3.2	24.2 ± 2.6
Handball No./week	1,9 ± 1	2.8 ± 0.6	1.0 ± 0
Handball activity h/week	3.1 ± 1.8	4.8 ± 1.1	1.5 ± 0
Experience handball	N.A.	12.65 ± 4.5	N.A.
Other Sports			
Fitness	11	4	7
Running	6	4	2
Volleyball	6	1	5
Yoga	4	4	0
Soccer	4	0	4
Gymnastics/Wheel Gymnastics	4	1	3
Swimming	3	1	2
Horse riding	3	1	2
Badminton	3	1	2
Track and field	2	0	2
Bouldering	1	1	0
Basketball	1	0	1
Tennis	1	0	1
Cheerleading	1	0	1
Table tennis	1	0	1
Sailing	1	0	1
Sports activity No./week	1.8 ± 1.3	1.5 ± 0.7	2 ± 1.5
Sports activity h/week	3.2 ± 2.6	2.4 ± 1.4	3.5 ± 3
KJOC-G Score D	90.7 ± 11.1	87.9 ± 13.8	93.6 ± 6.7
KJOC-G Score ND	94.9 ± 8.7	92.8 ± 11.3	97.0 ± 4.4

Data are given as mean and standard deviation (±) or as counts and percentages (%).

N.A., not applicable; KJOC-G, German version of the Kerlan-Jobe Orthopedic Clinic Shoulder and Elbow score; N/A, not applicable; D, dominant arm; ND, non-dominant arm.

### Effects of a handball-specific loading on the AHD

**Figure 1:**
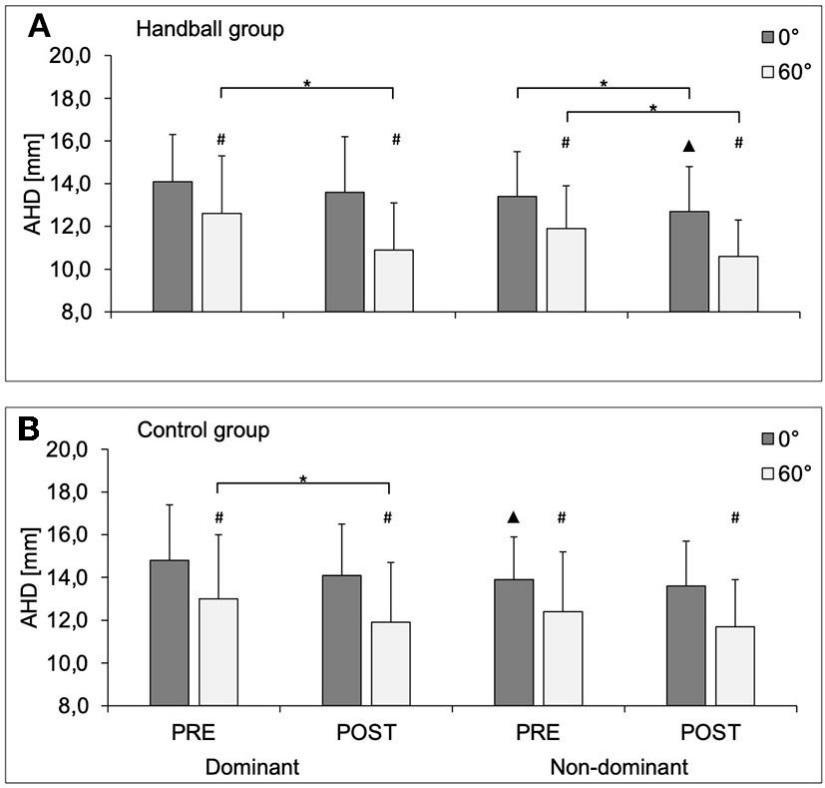
AHD (in mm) in 0° and 60° of abduction of the dominant and non-dominant shoulder in the handball group **(A)** and control group **(B)** before (PRE) and after (POST) a standardized handball protocol; AHD, Acromiohumeral distance; D, dominant shoulder; ND, non-dominant shoulder. * Significant difference between pre and post measurement; # significantly different to 0°; ▴significantly different to D (*P* values < 0.05).

Handball-specific loading led to reduced AHD in the dominant shoulder in the 60° abducted position in both groups (Figure 1) and in the non-dominant shoulder in 0° and 60° abducted position HB only.

In general, the elevation of the arm from 0° to 60° led to significantly shorter AHD (Figure 1). The AHD reduction with arm abduction was in general more pronounced post loading, while the difference was only significant in the dominant arm of HB (HB: Δ pre dominant −10.1 ± 14.9% and Δ post dominant −19.1 ± 11.7%; *p* = 0.018, d_RM_ = −0.53 Δ pre non-dominant −10.7 ± 12.3% and Δ post non-dominant −15.6 ± 11.5%; *p* = 0.098, d_RM_ = −0.38; CG Δ pre dominant −13.0 ± 10.6% and Δ post dominant −16.4 ± 11.6%; *p* = 0.202, d_RM_ = 0.44; Δ pre non-dominant −11.5 ± 13.3% and Δ post non-dominant −14.3 ± 10.9%; *p* = 0.288, d_RM_ = −0.23).

### AHD behavior in relation to shoulder strength and ROM

**Figure 2:**
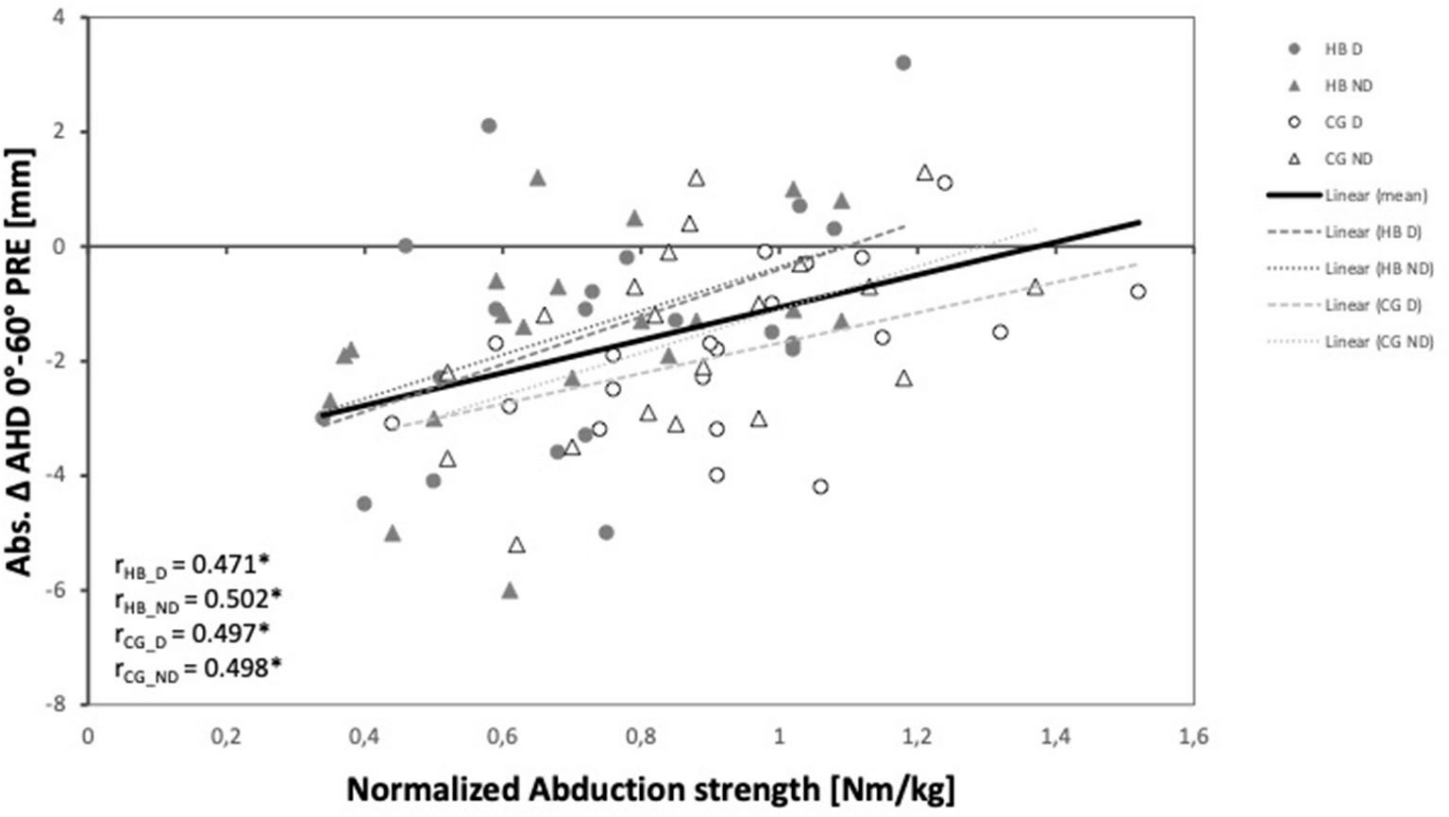
Relationship between absolute AHD change between 0° and 60° of abduction and normalized abduction strength of the dominant and non-dominant shoulder of both groups and the whole study population. AHD, Acromiohumeral distance; D, dominant shoulder; ND, non-dominant shoulder; HB, handball group; CG, control group.

A larger reduction of the AHD with arm abduction (0°➔ 60°; PRE) correlated moderately and significantly with smaller normalized abduction strength in both arms in both groups (Figure 2). No other generalizable effects on AHD, independent of shoulder side and study group, were detected.

### Effects of sport-specific experience

The subjectively perceived local fatigue in the dominant 'and non-dominant arm 'post handball-specific loading was significantly (*p* ≤ 0.05) higher in HB (Borg Scale dominant: 14.8 and non-dominant: 10.15) compared to CG (Borg Scale dominant: 13.2 and non-dominant: 8.8).

No significant interaction was detected between time and group for all positions and sides [0° dominant: F_(1.0, 38.0)_ = 0.09, *p* = 0.765, partial η^2^ = 0.00; 60° dominant: F_(1.0, 38.0)_ = 1.14, *p* = 0.292, partial η^2^ = 0.03; 0° non-dominant: F_(1.0, 38.0)_ = 0.69, *p* = 0.410, partial η^2^ = 0.02; 60° non-dominant: F_(1.0, 38.0)_ = 0.81, p = 0.375, partial η^2^ = 0.02].

HB and CG did not significantly differ in AHD absolute values (Figure 1) and ROM (Table 2). Furthermore, both groups did not significantly differ in AHD Δ Pre-Post 0° (HB: dominant −0.5 ± 1.5 mm; non-dominant −0.7 ± 1.5 mm; CG: dominant −0.7 ± 1.6 mm; non-dominant −0.3 ± 1.9 mm) and AHD Δ Pre-Post 60° (HB: dominant −1.7 ± 2.0 mm; non-dominant −1.3 ± 1.8 mm; CG: dominant −1.1 ± 2.0 mm; non-dominant −0.7 ± 2.4 mm).

**Table 2:** Strength, peak torque and range of motion of the dominant and non-dominant shoulder in the two groups.

	**Handball group**		**Control group**	
**Movement direction**	**D**	**ND**	**D**	**ND**
ABD-strength (N)	102.4 ± 40.0^a, b^	96.2 ± 37.9^b^	131.3 ± 43.3^a^	123.2 ± 40.0
ER-strength (N)	49.1 ± 11.3^a, b^	44.3 ± 11.0^b^	58.4 ± 15.1^a^	54.8 ± 13.4
IR-strength (N)	45.4 ± 11.0^b^	44.0 ± 9.8^b^	57.0 ± 19.3	54.6 ± 17.4
ER/IR strength ratio	1.09 ± 0.16^a^	1.01 ± 0.15^b^	1.07 ± 0.23	1.04 ± 0.17
Normalized ABD peak torque (Nm/kg)	0.75 ± 0.25^a, b^	0.70 ± 0.24^b^	0.94 ± 0.26^a^	0.88 ± 0.23
Normalized ER peak torque (Nm/kg)	0.17 ± 0.04^a, b^	0.15 ± 0.04^b^	0.20 ± 0.04^a^	0.19 ± 0.04
Normalized IR peak torque (Nm/kg)	0.16 ± 0.04^b^	0.15 ± 0.04^b^	0.20 ± 0.05	0.19 ± 0.05
ER-ROM (°)	98.9 ± 9.9^a^	94.8 ± 9.2	92.6 ± 12.4	91.1 ± 7.2
IR-ROM (°)	40.2 ± 12.1^a^	47.2 ± 13.6	46.7 ± 17.0	53.0 ± 16.6
TROM (°)	139.1 ± 12.8	142.0 ± 13.2	139.3 ± 19.3	144.1 ± 16.6
GIRD (°)	−7.0 ± 11.5		−6.3 ± 14.1	

Data are given as mean and standard deviation (±). D, dominant shoulder; ND, non-dominant shoulder; ABD, abduction; ER, external rotation; IR, internal rotation; ROM, range of motion; TROM, total range of motion; GIRD, glenohumeral internal rotation deficit. ^a^Significantly different to the non-dominant shoulder (*P* < 0.05). ^b^Significantly different to the control group (*P* < 0.05).

In terms of strength differences, CG showed significantly higher absolute ER, IR and ABD strength and normalized peak torque values in both shoulder sides and a larger ER/IR ratio in the non-dominant shoulder compared to HB (Table 2).

**Figure 3:**
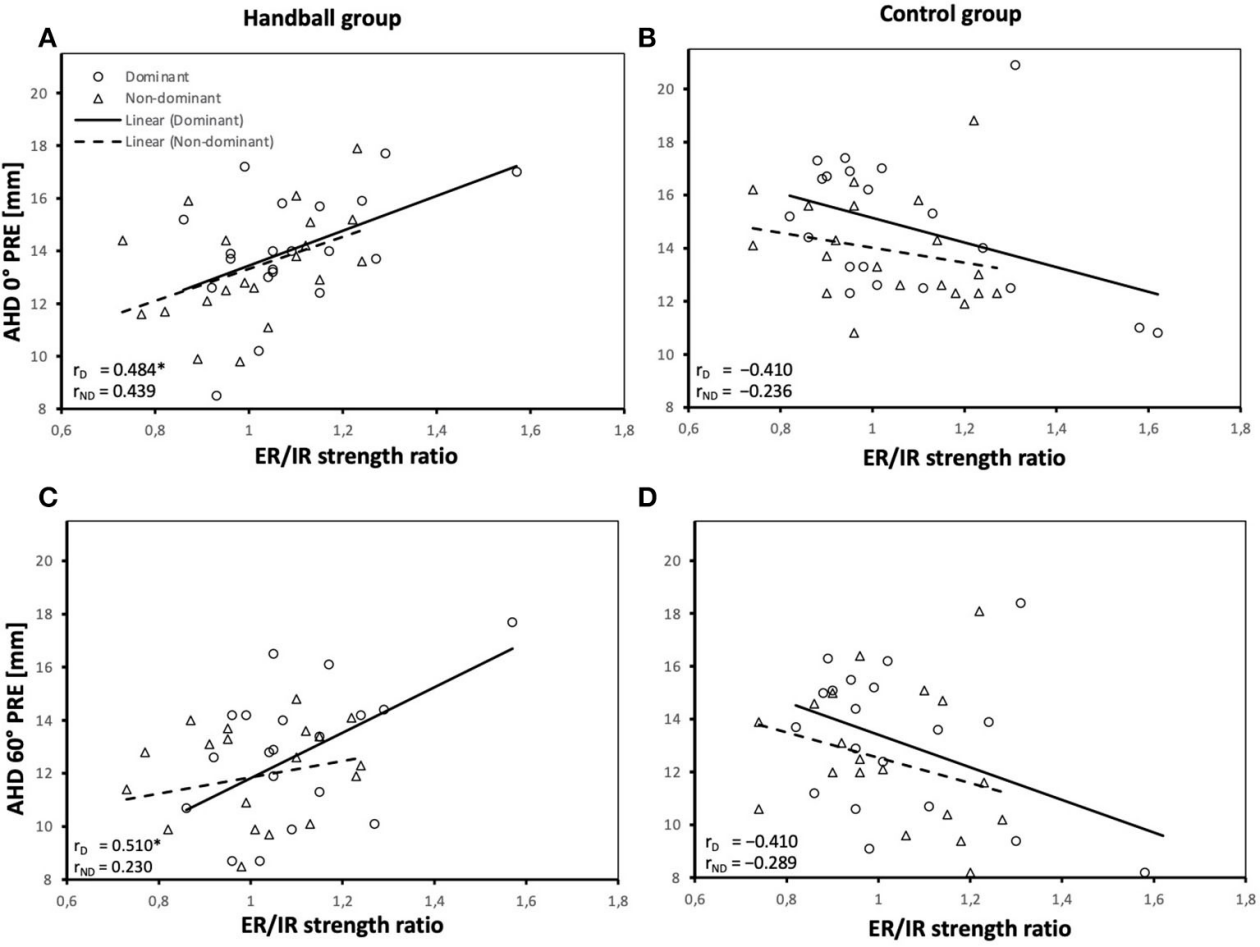
Relationship between ER/IR strength ratio and AHD pre-loading in 0° arm position **(A,B)** and AHD pre-loading in 60° abduction position **(C,D)** of the dominant and non-dominant shoulder of both groups. AHD, Acromiohumeral distance; D, dominant shoulder; ND, non-dominant shoulder; HB, handball group; CG, control group; ER, external rotation; IR, internal rotation. *Significant correlations (*P* values <0.05).

**Figure 4:**
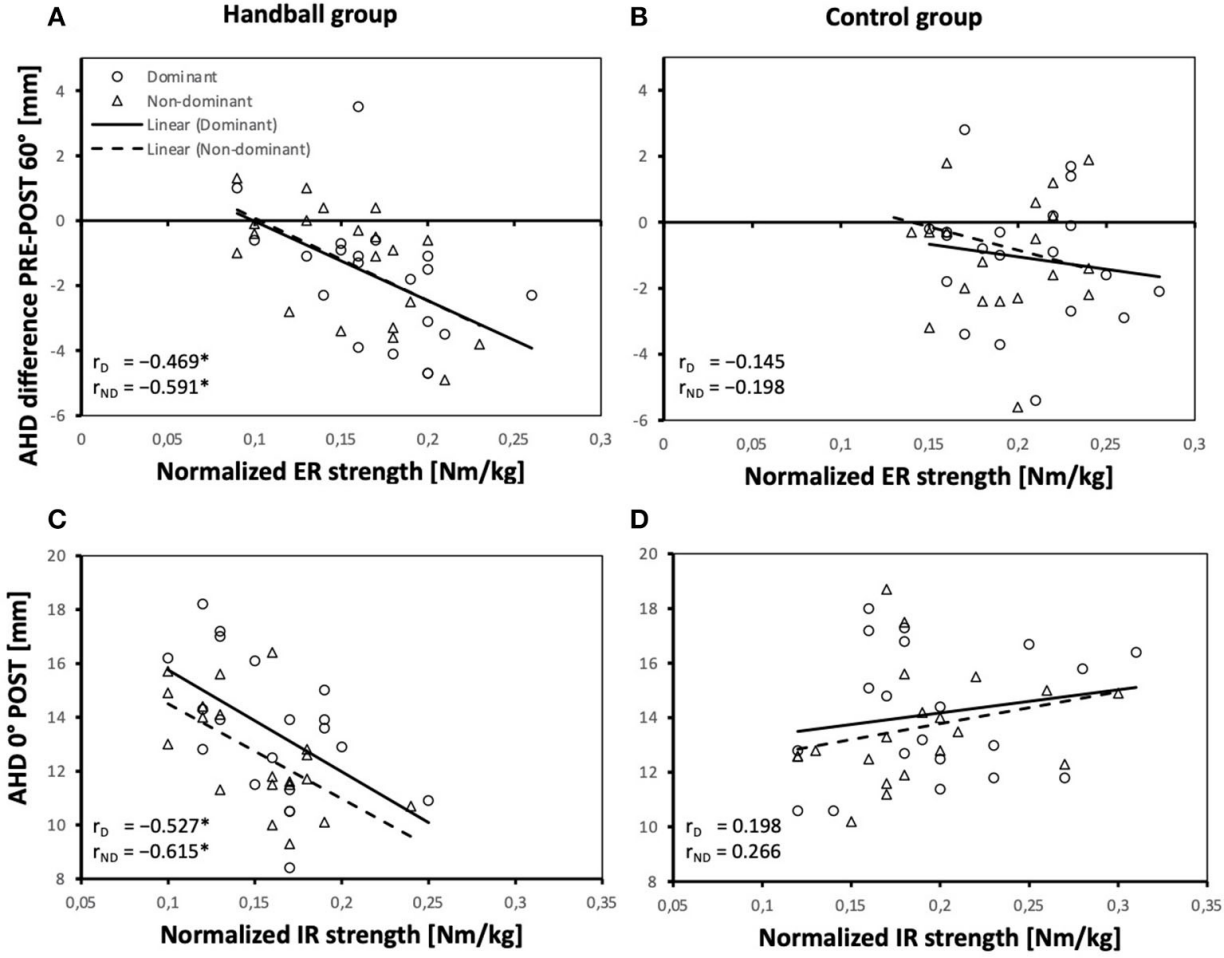
Relationship between the AHD difference pre-post loading in 60° abduction position and normalized ER strength of the dominant and non-dominant shoulder of both groups **(A,B)** and relationship between the AHD post-loading in 0° arm position and normalized IR strength of the dominant and non-dominant shoulder of both groups **(C,D)**; AHD, Acromiohumeral distance; D, dominant shoulder; ND, non-dominant shoulder; HB, handball group; CG, control group; ER, external rotation; IR, internal rotation. *Significant correlations (*P* values <0.05).

In terms of the effects of muscle strength on the AHD, the relationship of the ER/IR strength ratio and the AHD at 0° and 60° pre-loading (Figure 3) and the acute reactions of the AHD to handball-specific loading in 0° and 60° abducted shoulder positions differed between groups (Figure 4).

While in HB a larger AHD before loading was associated with a higher ER/IR strength ratio both at 0° and 60° with significant correlations detected for the dominant shoulder, this association was not detected in CG (Figure 3) and correlations differed significantly between CG and HB.

A larger AHD reduction with loading was associated with larger normalized ER strength in both shoulders (at 60°) in HB only (Figures 4A,B). Post loading, a larger AHD at 0° significantly correlated with smaller normalized IR strength in both shoulders of HB only (Figures 4C,D) and correlations differed significantly between CG and HB.

### Sex differences

Regarding anthropometric measures, males were displaying a significantly larger body height, mass, BMI, arm and forearm length. Furthermore, they displayed significantly larger KJOC-G scores for the non-dominant arm. Within CG, male athletes were older than female athletes.

ROM did not significantly differ between male and female participants. Absolute strength (ER, IR, ABD) and normalized peak torque (ABD) were significantly higher in male compared to female participants.

Absolute AHD were larger in male participants in both shoulder sides which was significant in all conditions, except for the AHD of the non-dominant shoulder in 0° (pre-measurement) and the AHD of the non-dominant shoulder in 0° and 60° (post-measurement).

## Discussion

To uncover how handball-specific loading acutely affects the subacromial space, we measured the AHD before and after a handball-specific loading protocol to provoke muscle fatigue that resembled handball-specific fatigue. At the same time, we wanted to determine the relation of effects with individual athlete characteristics and adaptations.

Handball-specific loading can aggravate AHD reduction, while the magnitude depends on sport-specific experience and arm abduction angle. Furthermore, shoulder abduction strength affects the maintenance of the AHD positively. In comparison to controls, experienced handball players showed less shoulder strength. Handball players displayed AHD reductions in both sides after fatigue, while in controls fatigue reduced the AHD in the dominant side only.

### Effects of a handball-specific loading on the AHD

We hypothesized that handball-specific loading will have negative effects on the AHD in experienced handball players and non-handball experienced athletes. Our hypothesis was confirmed as handball-specific fatigue led to an AHD reduction, especially when the arm was raised. This may constitute a potential injury risk of the structures located inside the subacromial space, e.g., the supraspinatus tendon.

Our results are in accordance with Chopp, O'Neill (41) who investigated humeral head translation with radiography in healthy men before and after a fatiguing task that simulated overhead job tasks and were intended to exhaust the rotator cuff. They showed that most of the participants displayed superior humeral head excursion as an effect of arm angle and fatigue and concluded that overhead work may accelerate the development of subacromial impingement by reduction of the subacromial space (41).

In contrast to our results, Maenhout et al. (23) found an increased AHD after fatigue in healthy recreational overhead athletes when the upper extremity was actively positioned at 45° or 60° of abduction. They assumed that the scapula compensated for shoulder-muscle fatigue. However, their fatigue protocol consisted of loaded overhead shoulder exercises until muscular fatigue and differed from our protocol which primarily intended to induce handball-specific loading. As our results show that AHD reduction was increased in participants (HB group) with higher subjective fatigue, it seems likely that, fatigue associated with handball specific loading may increase the risk of injury.

It maybe questioned to what extent the described AHD reduction of few millimeters from pre- to post-fatigue is clinically important. However, even a small reduction of the AHD may increase the pressure in the subacromial space and thus compromise inlying structures. As Girometti, De Candia (9) detected that in neutral position bursal and tendon tissues occupied approximately 44% of the subacromial space in overhead athletes and healthy controls, any reduction in the subacromial space, especially upon further arm elevation or after fatigue, could create a potentially injury-prone situation.

### AHD behavior in relation to shoulder strength and ROM

Our hypothesis that poor shoulder strength and poor range of motion will have negative effects on the ability to preserve the subacromial space can only be confirmed with regard to shoulder strength. Shoulder muscle strength affects the maintenance of the AHD. One goal should be appropriate training of the shoulder muscles so that the AHD is maintained as much as possible. It must be taken into account that it is not generally greater muscle strength that has a positive effect, but that the strength ratio between external and internal shoulder rotation is also important.

Since larger normalized abduction strength was related to smaller reduction of the AHD with arm abduction, we conclude that abduction strength contributes to the preservation of the AHD during arm lifting in non-fatigued conditions. The protective effect was observed independent of the investigated arm (dominant and non-dominant) and group (experienced and non-experienced), which points toward a general mechanism. Although the AHD is reduced during arm abduction, when the abductor muscles are active (14, 42), this does not mean that abductor muscle strength in general impacts the subacromial space negatively.

In contrast, the finding that poor abduction strength has a negative effect on maintaining the AHD suggests that it is relevant for athlete health to include abduction strength exercises into handball specific strength and conditioning training in order to prevent subacromial space reducing conditions that can lead to injuries (43).

In addition, the finding that experienced handball players examined in our study showed lower shoulder strength and reported higher fatigue in the dominant arm than the non-handball experienced athletes suggests that additional strength training in handball is indispensable and should be expanded. Upper extremity strength training can be easily and inexpensively implemented in regular handball training (e.g., with elastic resistance bands) as previous studies have already shown (44, 45).

### Effects of sport-specific experience

We hypothesized that handball-specific experience and therewith associated adaptations may affect the response of the AHD to handball-specific loading. Our hypothesis was confirmed since experienced handball players and not-handball experienced athletes showed different effects after handball-specific loading. Although the handball players show handball-specific adaptations, this does not necessarily lead to a better preservation of the AHD and thus no better injury protection.

Our results do not confirm protective, impingement-sparing conditions in overhead athletes as detected by Maehnout et al. (23). Handball players in our study showed less shoulder strength and did not better preserve the AHD than controls. On the contrary, they showed AHD reductions in both arms after fatigue while in controls the AHD was significantly reduced in the dominant arm only.

In experienced handball players the absolute AHD and its reduction after fatigue are related to strength and strength ratios differently than in controls. A larger ER/IR ratio was associated with a larger AHD in handball players in the pre-fatigue state. Larger ER and smaller IR strength abilities appear to be conducive to maintain the AHD. After fatigue, the handball players with greater IR strength showed a smaller AHD in the hanging arm, which corresponds to the previous result. However, the change through fatigue in terms of AHD reduction was greater in those with greater ER strength. This might be due to the larger space for reduction.

Nevertheless, these results suggest that greater ER strength has a positive effect on preserving the subacromial space, which is why strengthening the external rotators should be taken into account in the training of handball players.

Since this association was not detected in non-handball experienced athletes a disparity in ER/IR strength might affect the AHD only in connection with other sport specific adaptations which have not been identified within this study. This fact illustrates the effect of interpersonal differences related to the investigated groups, which might be caused by handball-specific experience and morphological adaptations.

### Sex differences

We found larger AHD values in male participants, which has already been documented in previous studies (46). However, in agreement with our hypothesis the effects of handball-specific loading on AHD and the relation between AHD and intrinsic factors did not differ between sexes. This leads to the conclusion that interventions in terms of shoulder injury prevention can be developed independent of sex.

### Limitations

Since this study investigated athletes with healthy shoulders only, our assumptions cannot be extended to athletes suffering from shoulder pathologies.

ER and IR strength testing was performed in in 90° abduction position of the shoulder, as this is the sport specific position for throwing movements. However, this position may have an effect on force development, so that comparisons with other studies and positions must be made with caution.

Although our standardized handball-specific loading protocol resembled the characteristic load of handball players, the intensity of movement executions and movement patterns could differ in real gameplay. Since our loading protocol resulted in individualized fatigue, it is possible that participants within a group experienced different levels of fatigue. Although subjective fatigue was measured using the Borg scale, differences in heart rate or distance covered may have occurred and may have affected the results.

## Conclusion

Handball-specific fatigue leads to an AHD reduction which is enhanced with shoulder abduction. Furthermore, poor abduction strength affects the preservation of the AHD negatively in both handball experienced and not experienced athletes, which may increase the risk for shoulder injuries. In handball players, greater ER and ABD strength had a positive effect on the maintenance of the AHD. Therefore, strengthening of external rotator and abductor muscles should be an important part in handball training.

## Data availability statement

The raw data supporting the conclusions of this article will be made available by the authors, without undue reservation.

## Ethics statement

The studies involving human participants were reviewed and approved by Ethics Committee of the Faculty of Humanities and Social Sciences of the Humboldt-Universität zu Berlin (HU-KSBFEK_2019_0013). The patients/participants provided their written informed consent to participate in this study.

## Author contributions

CR and KL contributed to conception, design of the study, and performed the statistical analysis. CR conducted the data collection and wrote the first draft of the manuscript. All authors contributed to manuscript revision, read, and approved the submitted version.

## Funding

This study is part of the PhD project of CR, which is partially funded by a PhD scholarship of the FAZIT-Foundation of the Frankfurter Allgemeine Zeitung, Frankfurt am Main, Germany. The article processing charge was funded by the Deutsche Forschungsgemeinschaft (DFG, German Research Foundation) - 491192747 and the Open Access Publication Fund of Humboldt-Universität zu Berlin.

## Conflict of interest

The authors declare that the research was conducted in the absence of any commercial or financial relationships that could be construed as a potential conflict of interest.

## Publisher's note

All claims expressed in this article are solely those of the authors and do not necessarily represent those of their affiliated organizations, or those of the publisher, the editors and the reviewers. Any product that may be evaluated in this article, or claim that may be made by its manufacturer, is not guaranteed or endorsed by the publisher.
