# Supplementary material for: Handball-specific loading acutely reduces the acromiohumeral distance in experienced handball players and in non-handball experienced athletes
Source: Front Sports Act Living. 2022 Sep 16;4:997401. doi: 10.3389/fspor.2022.997401 (PMC9524289; doi:10.3389/fspor.2022.997401)
Supplement: Supplementary file 1 [file Data_Sheet_1.PDF]

Handball-specific loading protocol
(Simulating shoulder demands during handball match)

Warm-up: athletes will be asked to carry out an individual handball-specific warm-up of 20 min in pairs consisting of the following exercises:

Exercises	Repetitions/Duration
**1) General warm-up**	
Running	Minimum 5x20 m
Sprinting	Minimum 3x20 m
Bodyweight exercises:<br>push-ups<br>plank	 Minimum 10 x<br>Minimum 60 sek
Shoulder stretching<br>(e.g. arm circling)	Upon need
**2) Handball-specific warm-up**	
Passing<br>Short distance (5 m)<br>Long distance (20 m)	 20x<br>10x
Shots on goal<br>Jump shots<br>Set shots	 5x<br>5x
Tackles (attack-defense exercise)<br>Attacks<br>Defense	 5x<br>5x

Standardized Handball-specific loading protocol:

- The loading protocol consists of four parts which are completed in pairs, are carried out in the given order and in quick succession (max. rest time between the exercise blocks: 1 min)
- All shots are taken at a goal with a goalkeeper to ensure a realistic game/training setting

Exercises	Repetitions
**1) Passing**	
Passes short distance (5m)	60x
Passes long distance (20m)	20x
**2) Shots on goal**	
Jump shots (9m)	3x
Set shots (9m)	3x
Penalty shots (7m)	3x
**3) Tackles**	
Tackles - Attacks	9x
Tackles - Defense	9x
Tackles - Attacks	9x
Tackles - Defense	9x
**4) Defense Blocking**	
Blocking of shots	4x

Setting and material:

- Sports facility with official handball court (20m x 40m)
- Official handball goal (2 x 3m)
- Handball: official International Handball Federation (IHF) team handball: size 2 (female), size 3 (male)
